# Attributes of leadership skill development in high-performance pre-hospital medical teams: results of an international multi-service prospective study

**DOI:** 10.1186/s13049-024-01221-1

**Published:** 2024-05-21

**Authors:** J. A. Deodatus, M. A. Kratz, M. Steller, N. Veeger, B. Dercksen, R. M. Lyon, M. Rehn, L. Rognås, C. Coniglio, B. Sheridan, C. Tschautscher, D. J. Lockey, E. ter Avest

**Affiliations:** 1 Department of Acute Care, University Medical Center Groningen, University of Groningen, Groningen, the Netherlands; 2 Emergency Medical Services, Centre for Prehospital Emergency Care, Department of Emergency, Anaesthesia and Pain Medicine, FinnHEMS 30 & 40, Tampere University, Tampere, Finland; 3 Department of Epidemiology, Medical Centre Leeuwarden, Leeuwarden, The Netherlands; 4 Mobile Medical Team (MMT), Lifeliner 4, Eelde, the Netherlands; 5 Department of Anaesthesiology, University Medical Center Groningen, University of Groningen, Groningen, the Netherlands; 6 Air Ambulance Kent Surrey Sussex & Department of Health Sciences, University of Surrey, Redhill, UK; 7 Air Ambulance Department, Division of Prehospital Services, Oslo University Hospital, Oslo, Norway; 8 Department of Research and Development, Norwegian Air Ambulance Foundation, Oslo, Norway; 9 Institute of Clinical Medicine, University of Oslo, Oslo, Norway; 10 The Danish Air Ambulance, Brendstrupgårdsvej 7, 2. Th, 8200 Aarhus N, Denmark; 11 Department of Anesthesia, Intensive Care and Pre-Hospital Emergency Medical Services, Maggiore Hospital Carlo Alberto Pizzardi, Bologna, Italy; 12 Department of Anaesthesia and Hunter Retrieval Service, John Hunter Hospital, Lookout Rd, New Lambton Heights, NSW 2305 Australia; 13 Department of Emergency Medicine, School of Medicine and Public Health, UW Health Med Flight and Berbee Walsh University of Wisconsin, 600 Highland Avenue, Madison, WI 53792 USA; 14 London’s Air Ambulance and Bart’s Health NHS Trust, Royal London Hospital, London, E1 1FR UK

**Keywords:** Non-technical skills, Leadership, Training, Helicopter emergency medicine

## Abstract

**Backgrounds:**

Team leadership skills of physicians working in high-performing medical teams are directly related to outcome. It is currently unclear how these skills can best be developed. Therefore, in this multi-national cross-sectional prospective study, we explored the development of these skills in relation to physician-, organization- and training characteristics of Helicopter Emergency Medicine Service (HEMS) physicians from services in Europe, the United States of America and Australia.

**Methods:**

Physicians were asked to complete a survey regarding their HEMS service, training, and background as well as a full Leader Behavior Description Questionnaire (LBDQ). Primary outcomes were the 12 leadership subdomain scores as described in the LBDQ. Secondary outcome measures were the association of LBDQ subdomain scores with specific physician-, organization- or training characteristics and self-reported ways to improve leadership skills in HEMS physicians.

**Results:**

In total, 120 HEMS physicians completed the questionnaire. Overall, leadership LBDQ subdomain scores were high (10 out of 12 subdomains exceeded 70% of the maximum score). Whereas physician characteristics such as experience or base-specialty were unrelated to leadership qualities, both organization- and training characteristics were important determinants of leadership skill development. Attention to leadership skills during service induction, ongoing leadership training, having standards in place to ensure (regular) scenario training and holding structured mission debriefs each correlated with multiple LBDQ subdomain scores.

**Conclusions:**

Ongoing training of leadership skills should be stimulated and facilitated by organizations as it contributes to higher levels of proficiency, which may translate into a positive effect on patient outcomes.

**Trial registration:**

Not applicable.

**Supplementary Information:**

The online version contains supplementary material available at 10.1186/s13049-024-01221-1.

## Background

When treating critically ill patients, physicians working in high-performance teams such as trauma-, resuscitation-, or Helicopter Emergency Medicine Service (HEMS) teams need to be proficient in a wide range of clinical skills. These comprise both technical skills, such as advanced airway management and surgical procedures, and non-technical skills (NTS) such as effective crew resource management (CRM), communication, decision-making, and leadership [1].

Mastering non-technical skills is particularly important for HEMS physicians, who often have to manage teams made up of various Emergency Medical Services (EMS) providers only formed ad hoc on scene, while providing effective care for critically ill patients, often under challenging conditions. Team leadership is a particularly important non-technical skill as it allows to set priorities, to make decisions and to coordinate care in an efficient way, with different leadership behaviours being important in the initiation- and the maintenance phases of the resuscitation of critically ill patients [2]. Previous studies in high acuity medical situations such as cardiopulmonary resuscitation and trauma care consistently indicate that strong leadership skills are associated with improved outcomes [3–8].

Hence, many HEMS organizations and hospitals alike emphasize the importance of leadership skills during their recruitment process, and provide leadership training, which may include formal licenced courses or on-base scenario training using simple mannequins [2, 4, 9–12]. However, it is unknown how development of these skills can best be facilitated in order to improve proficiency.

In the present study, we aim to investigate how various subdomains (behaviors) of leadership have developed within an international cohort of HEMS physicians, and how these behaviors relate to physician, organization- and training characteristics.

## Methods

### Study design

An international multi-service prospective cross-sectional survey study was conducted to analyze leadership skills of physicians from different HEMS services in Europe, the United States of America (USA) and Australia. Leadership skills were evaluated using the self-reported version of the Leadership Behavior Development Questionnaire (LBDQ XII, see below), and the scores were correlated with physician-, organization- and training characteristics. LBDQ results and other data were collected anonymously through an electronic survey using the REDCap electronic data capture tool (version 12.4.6) hosted at the University Medical Centre Groningen (UMCG) [13, 14].

### Study population

Survey participants were HEMS physicians working at least 0.2 full-time equivalent in the prehospital environment, with no conditions on years of experience, base specialty or prior NTS training. HEMS physicians in training (pre- sign-off) were excluded from participation. A minimum response target number was set at 50 participants based on previous studies investigating leadership in high-acuity medical settings [6, 15]. To ensure a sufficiently heterogeneous cohort, physicians were recruited from 8 different HEMS organizations on 3 continents.

### Data acquisition

Data were collected via a 3-part electronic survey from June 2023 to August 2023. In the first part of the survey, a comprehensive set of physician-, organization- and training characteristics were gathered that were hypothesized to correlate with leadership development within the HEMS context. In the second part, leadership skills were assessed using the the LBDQ XII self-assessment questionnaire (see below). At the end of the questionnaire participants were asked which leadership subdomain they felt they could most improve on, and how they thought this could be best achieved. Participants were prompted to answer all the questions, before they could continue with a next page in the survey. The full survey is available in the supplementary data, Appendix A.

Collected physician characteristics included age, nationality, gender, working experience and base specialty, while organization characteristics were garnered from questions regarding standards for NTS/CRM-training, on-base training facilities and mission-debriefs. Questions on training characteristics elicited details about NTS/CRM training during the organization’s induction period as well as participation in formal courses.

The LBDQ XII self-assessment questionnaire contains 100 statements about leadership that are rated by the participant on a five-point Likert-type scale ranging from “always” to “never” [16, 17]. Each statement corresponds to one of 12 specific leadership behaviors (subdomains) as outlined in Table 1. LBDQ subdomain scores were calculated from the answers given by each participant. Subdomain scores were subsequently represented as percentages of the maximum score for that particular subdomain, to enable comparison of scores across subdomains (maximum scores differed).

**Table 1:** LBDQ Leadership subdomains

LBDQ subdomains	Explanation
1. Representation	Does the leader speak as the representative of the group?
2. Reconciliation	Does the leader reconcile conflicting demands, and does he/she reduce disorder to system?
3. Tolerance of uncertainty	Is the leader able to tolerate uncertainty and postponement without anxiety or getting upset?
4. Persuasion	Does the leader use persuasion and argument effectively, and does he/she exhibit strong convictions?
5. Initiation of structure	Does the leader clearly define his own role, and does he/she let followers know what is expected?
6. Tolerance and freedom	Does the leader allow followers scope for initiative, decision, and action?
7. Role assumption	Does the leader actively exercise the leadership role rather than surrendering leadership to others?
8. Consideration	Does the leader regard the comfort, well-being, status, and contributions of followers?
9. Production emphasis	Does the leader apply pressure for productive output?
10. Predictive accuracy	Does the leader exhibit foresight and ability to predict outcomes accurately?
11. Integration	Does the leader maintain a closely-knit organization; does he/she resolve inter-member conflicts?
12. Superior orientation	Does the leader maintain cordial relations with superiors, and does he/she have influence with them/is striving for higher status?

*LBDQ* leadership subdomains, as summarized from Stogdill (1963) [17]

### Outcome definitions

The primary outcome was defined as the various leadership subdomain scores of HEMS physicians measured with the Leader Behavior Description Questionnaire (LBDQ).

Secondary outcomes were:

1. The association of LBDQ scores with specific physician-, organization- and training characteristics.
2. Self-reported ways to improve leadership skills.

### Ethical considerations

Informed consent was obtained from all participants prior to data collection. All data was collected anonymously and untraceable to individual physicians. This study was approved by the Medical Ethical Committee of the UMCG (METc 2022.604).

### Analyses

Due to survey-prompting, there was no missing data in returned surveys. Characteristics of participating physicians are reported as mean (SD) or median (IQR) and numbers (%). Normality of data was evaluated through Shapiro–Wilk tests and visual inspection using frequency histograms and Q-Q plots. Univariate linear models were constructed to examine the relationship between physician-, organization- and training characteristics and each of the leadership subdomain scores. Subsequently, linear mixed-effects modelling (LMEM) was employed with stepwise approach to identify independent predictors of leadership subdomain scores. Predictors demonstrating statistical significance in univariate analysis (*p* < 0.05) were entered as fixed effects and country of origin entered as a random effect, as this was considered a non-modifiable factor with potential effect on leadership scores. In stepwise regression analysis, both forwards and backwards selection methods were employed for model optimization. The Bayesian Information Criterion (BIC) was used for model selection. Finally, partial R-squared values were calculated to assess the unique contribution of each predictor to the explained variance in the model. Analyses were conducted using R, Rstudio, and the dplyr, ggplot2, MuMIn, glmmTMB, lme4 software packages [18–20].

## Results

### Participants

A total of 120 HEMS physicians completed the survey including the full LBDQ. Baseline characteristics of study participants are represented in Table 2. Median age of participants was 45 years (IQR 39–49). The majority were male (*n* = 89, 74.2%), and participants had on average 9 years of HEMS experience (IQR 5–14) Most physicians had a background in anesthesiology (*n* = 39 32.5%) or emergency medicine (*n* = 40, 33.3%). Respondents worked in eight different countries across Europe, Australia, and the USA. Clinical team composition and the number of patients attended on a yearly basis varied significantly across services.

**Tabel 2:** Characteristics of the study population (*n* = 120)

*Characteristics*	*Participants *(*N* = 120)
Male	89 (74.2%)
Age (in years)	45 (39–49 IQR)
Years of experience as a physician	17 (13–22 IQR)
Years of experience working in a pre-hospital environment	9 (5–14 IQR)
Base specialty (multiple base specialties is a possibility)	
Emergency medicine	40 (33.3%)
Anesthesiology	39 (32.5%)
Other (including combinations)	41 (34.2%)
Country of work	
Australia	13 (10.8)
Denmark	7 (5.8)
Finland	20 (16.7)
Italy	27 (22.5)
Netherlands	4 (3.3)
Norway	11 (9.2)
UK	20 (16.7)
USA	18 (15.0)
Hours of clinical HEMS work per week	15 (12–24 IQR)
Number of patients treated per week per HEMS physician	
0–5 patients	75 (62.5)
5–10 patients	31 (25.8)
10–15 patients	10 (8.3)
> 15 patients	4 (3.3)
Clinical team consistency	
2 person: doctor + paramedic/nurse	49 (40.8%)
3 person: doctor + 2 paramedic(s)/nurse(s)	5 (4.2%)
3 person: doctor + paramedic + pilot	34 (28.3%)
3 person: 2 doctors + 2 paramedic(s)/nurse(s)	1 (0.8%)
4 person: doctor + paramedic + 2 pilot(s)	9 (7.5%)
other	22 (18.3%)

*HEMS* Helicopter Emergency Medical Service, *UK* United Kingdom, *US* United States of America

### Primary outcome

**Fig. 1:**
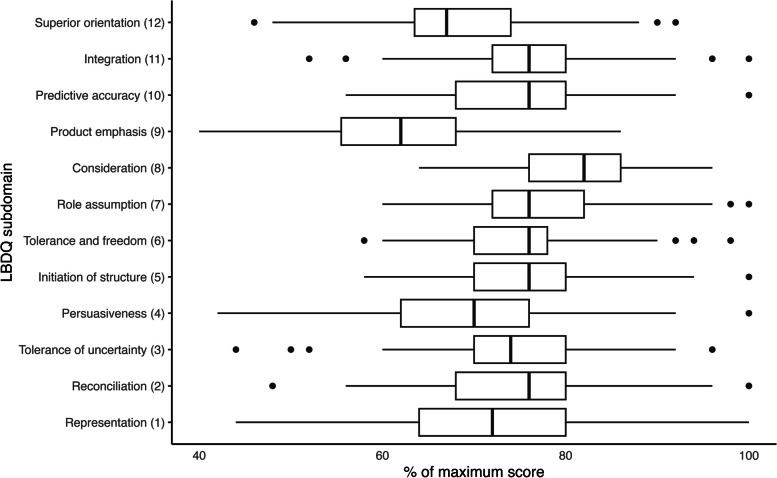
Leadership behavior scores (as percentage of the maximum score) of 12 subdomains of the LBDQ (*n* = 120). Boxplot of LBDQ subdomain scores depicting the median, IQR and range. Outliers are shown as dots

The average median subdomain score was 73,6% of the maximum score. Participants scored highest on consideration (median score 82%, IQR 76–86), role assumption (median 76%, IQR 72–82) and reconciliation (median 76%, IQR 68–80). The lowest score was found for production emphasis (median 62%, IQR 55.5–68). An overview of the LBDQ subdomain scores is shown in Fig. 1.

### Association of LBDQ scores with specific physician-, organization- or training characteristics

In univariate analyses, 18 different physician, service, and training characteristics were correlated to one or more of the 12 LBDQ leadership subdomains (Supplementary material, Appendix B). Ten of these characteristics remained independently associated after multivariate modelling with *r*-values ranging from 0.18–0.29 (Table 3).

**Table 3:** Physician-, service- and training characteristics independently associated with one or more leadership behaviors (*n* = 120)

**Physician characteristics**	**R**	**Service characteristics**		**R**		**Training characteristics**	**R**
Age		Operational	Mission debrief at unit		During induction	Observed clinical work	
**Reconciliation (2)**	**-.22**		**Tolerance of uncertainty (3)**	**.29**		**Persuasiveness (4)**	**.19**
						**Production emphasis (9)**	**.24**
			Standards for scenario training frequency at unit			**Superior orientation (12)**	**.21**
						Training of NTS/CRM	
			**Persuasiveness (4)**	**.20**		**Initiation of structure (5)**	**.20**
			**Consideration (8)**	**.26**		**Predictive accuracy (10)**	**.26**
		During recruitment	Evaluation of NTS/CRM			**Integration (11)**	**.18**
			**Persuasiveness (4)**	**.21**		Other induction content	
			Evaluation of leadership skills			**Structure initiation (5)**	**-.25**
					Other	Frequency formal	
			**Reconciliation (2)**	**.22**		NTS/CRM training	
			**Tol. Of uncertainty (3)**	**.24**		**Reconciliation (2)**	**.20**
			**Initiation of structure (5)**	**.26**		**Role assumption (7)**	**.22**
						Formal NTS/CRM training targeted leadership	
						**Tolerance and freedom (6)**	**-.26**

Represented are *r*-values for characteristics (in bold) independently associated with LBDQ leadership subdomains after linear mixed-effects modelling (LMEM) with stepwise regression with country of practice entered as a random effect

#### Physician characteristics

**Fig. 2:**
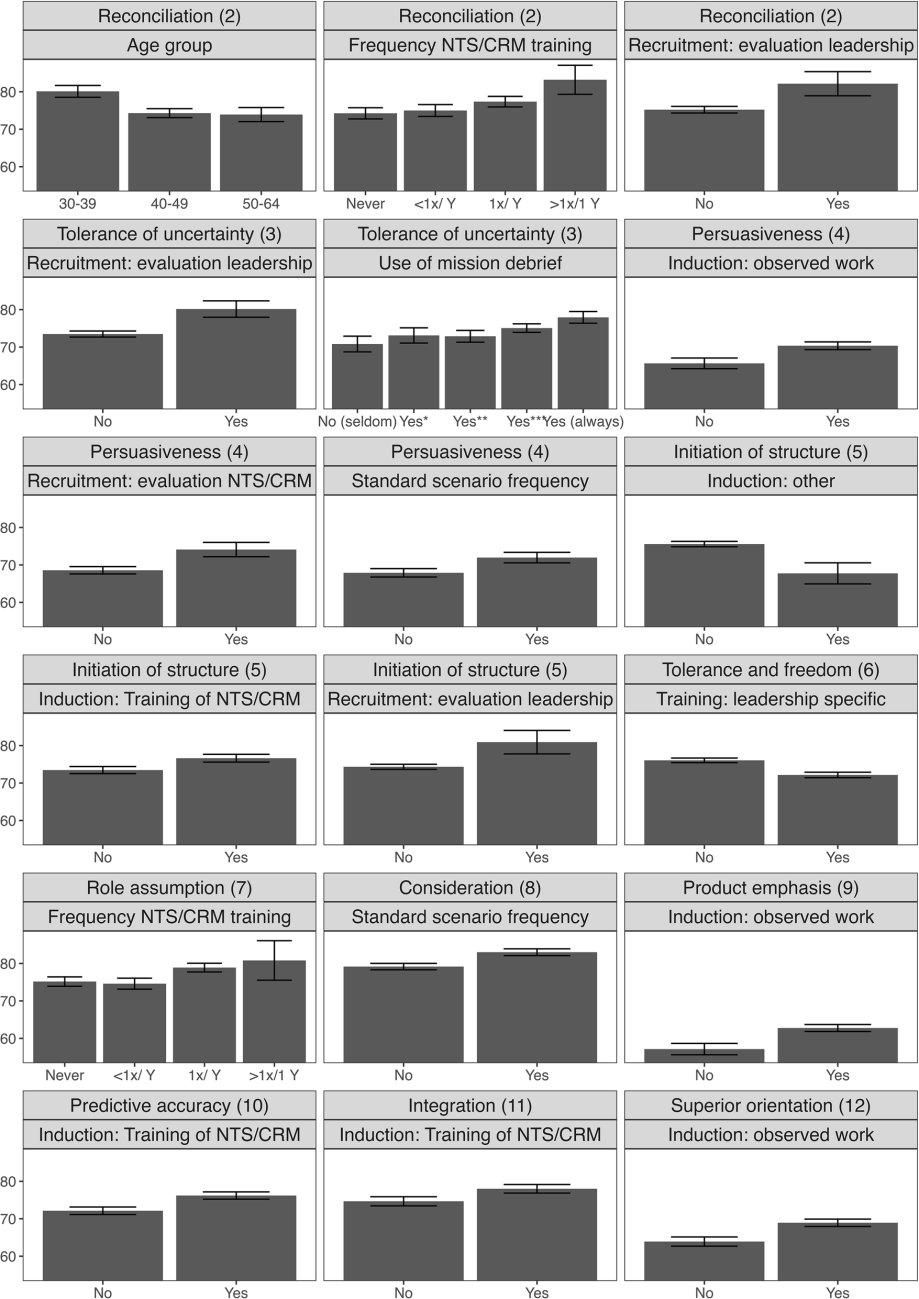
LBDQ subdomain scores as percentage of maximum scores (*n* = 120). Represented are mean (SE) percentages of LBDQ scores for independently correlated characteristics. NTS; non-technical skills; CRM, crew resource management

Physician age was associated with “reconciliation” scores. Younger physicians scored higher, representing a better ability to reconcile conflicting demands than older physicians (Fig. 2). Physician experience and base specialty were not significantly associated to any of the LBDQ subdomains.

#### Organizational characteristics

Physicians working for a HEMS service where NTS/CRM and leadership skills were specifically evaluated during the recruitment process were found to have higher scores in the LBDQ subdomains “persuasiveness”, “reconciliation”, “tolerance of uncertainty” and “initiation of structure”. When a HEMS organization had set standards for (the frequency of) scenario-based training, physicians scored higher on the subdomains of “persuasiveness” and “consideration”. When mission debriefs were part of the daily routine of the service, this was associated with higher scores for “tolerance of uncertainty” (Fig. 2).

#### Training characteristics

Specific training in NTS/CRM skills during the service induction period was positively associated with three subdomains: “structure initiation”, “production emphasis”, and “integration”. Also, observation of clinical work during the induction period showed positive association with “persuasiveness”, “production emphasis”, and “superior orientation”. A negative association was found for the relation between formal NTS/CRM training and the “tolerance of freedom” subdomain of the LBDQ.

### Self-reported ways to improve leadership skills in HEMS physicians

Participants were also asked which LBDQ subdomain they thought they could most improve on (Appendix C). Answers were diverse and all 12 subdomains were mentioned several times with reconciliation (14.2%), tolerance of uncertainty (10.8%) and role assumption (14.2%) mentioned most frequently. Physicians who indicated a need for improvement in a specific subdomain often (i.e. for 10 of the 12 subdomains mentioned) had lower median scores for those subdomains compared to those who did not express a need for improvement (data not shown).

## Discussion

In this study we found that HEMS physician’s leadership skills were overall well developed, irrespective of physician characteristics. Attention for- and training of these skills, by the organization, during recruitment and after induction into the service contributed to higher levels of proficiency. Although this study was carried out amongst HEMS physicians, our findings can likely be extrapolated to a wider cohort of critical care physicians, as leadership skills are of similar importance when leading in-hospital multidisciplinary critical care teams, such as resuscitation- and trauma teams.

As this is the first study to systematically assess leadership skills in a large international cohort of HEMS physicians using a validated questionnaire, it is difficult to value absolute scores for the various subdomains as reported. Although any comparison with historical data is likely hampered by substantive differences between professions, zeitgeist and self-reporting bias, the scores found in our cohort did align with those observed in other high-performance professions such as military personnel and aircraft operators [17, 21]. HEMS physicians scored highest on the specific leadership behavior “consideration”, which is regarded as one of the principal indicators of effective leadership [3, 22]. The lowest scores were reported for the “production emphasis” and “superior orientation” subdomains. This may be explained by the specific context of HEMS operations: crews typically function as small, well-established teams that inherently maintain a collective, outcome-oriented focus. In such settings, the responsibility for initiating and maintaining this focus may fall less on the leading physician and more on cohesive team dynamics. Also, physicians in these teams are generally self-proficient on scene, prioritizing adaptive leadership and teamwork over superior orientation.

Health care organizations, and in particular HEMS services seem to be aware of the importance of leadership skills, as they often mention these skills as required- or desired attributes when they recruit [23]. In this study we found that physicians recruited into organizations where specific attention was given to leadership skills during the recruitment process on average scored higher on four specific leadership subdomains (“persuasiveness”, “reconciliation”, “tolerance of uncertainty” and “initiation of structure”). Hence, our findings indicate that services are able to evaluate leadership skills early during the recruitment process for their organizations, and are able to select the best suited candidates possessing the desired skills.

After commencing employment, training- and organization characteristics play a pivotal role in the further development of leadership skills: Specific attention to leadership skills during service induction contributed to higher scores on 6 of the 12 leadership subdomains. Organization-specific attributes such as systematic mission debriefs (previously mentioned as a means to improve patient outcomes [24]) and on-base scenario training were associated with higher leadership scores. Both processes may have a direct impact on leadership skills by reinforcing a shared mental model that emphasizes the importance of leadership as a component of effective teamwork. These results highlight the significance of training as a crucial factor for predicting and improving leadership skills and are in line with previous literature [4, 8, 12, 22, 25]. Of note, the observed positive effect of leadership skills training was independent of team composition, team size, base specialty, patient exposure or years of experience, as none of these factors were related to any of the 12 leadership subdomain scores. Thus, ongoing training in leadership skills may be beneficial, irrespective of level of experience. Interestingly, while most training characteristics were positively correlated to leadership behavior, physicians who attended formal NTS/CRM training scored lower on “tolerance of freedom”. In the context of HEMS, this may be explained by an emphasis on structured, directive leadership, adherence to protocols, and reduced individual autonomy in specific leadership-oriented NTS/CRM training. This finding aligns with previous research which suggests that high-acuity settings often require a more directive leadership style [26].

Our findings not only emphasize the importance of (facilitating) training, they also demonstrate that targeted training, tailored down to an individual level, may be possible. As the reported association between various LBDQ subdomain scores and self-reported potential areas of improvement suggests that individual HEMS physicians are aware of potential areas for improvement in their own leadership skills. Training can be directed specifically aimed at these area’s.

Despite the large, international and heterogenous study population, our study had several limitations. First, self-scoring (even when using a validated questionnaire) risks social-desirability bias. Therefore, our findings may have overestimated HEMS physician leadership skills. However, given the context of this study, where peer-rating is difficult, we believe this is largely unavoidable. Second, most associations between the various physician, service, and training characteristics and the various leadership subdomains were weak, each explaining less then 10% of observed variance. In this respect, it is worth recognizing that quantitative assessment of soft skills, like leadership, inherently involves complexities and nuances not fully captured by traditional statistical measures. Further, concerns regarding multiple hypothesis testing may arise due to the substantial number of correlation tests performed. However, this was largely unavoidable as a priori all twelve subdomains were deemed equally important. Lastly, it is difficult to state to which extent the reported variance in leadership scores as reported in relation to the variables explored translates into clinically relevant differences, especially as subdomain scores were high for the group as a whole.

## Conclusion

Ongoing training of leadership skills should be stimulated and facilitated by organizations as it contributes to higher levels of proficiency, which may translate into a positive effect on patient outcomes.

### Supplementary Information

Supplementary Material 1.

Supplementary Material 2.

Supplementary Material 3.

## Data Availability

The datasets used and/or analysed during the current study are available from the corresponding author on reasonable request.

## Abbreviations

BIC: Bayesian Information Criterion
CRM: Crew Resource Management
EMS: Emergency Medical Services
HEMS: Helicopter Emergency Medical Services
IQR: Interquartile range
LBDQ: Leader Behavior Description Questionnaire
LMEM: Linear Mixed Effects Modeling
NTS: Non-technical skills
