# Supplementary figures and images for: Attributes of leadership skill development in high-performance pre-hospital medical teams: results of an international multi-service prospective study
Source: Scand J Trauma Resusc Emerg Med. 2024 May 21;32:46. doi: 10.1186/s13049-024-01221-1 (PMC11107030; doi:10.1186/s13049-024-01221-1)

# **Appendix A:** Questionnaire including LBDQ-form XII self


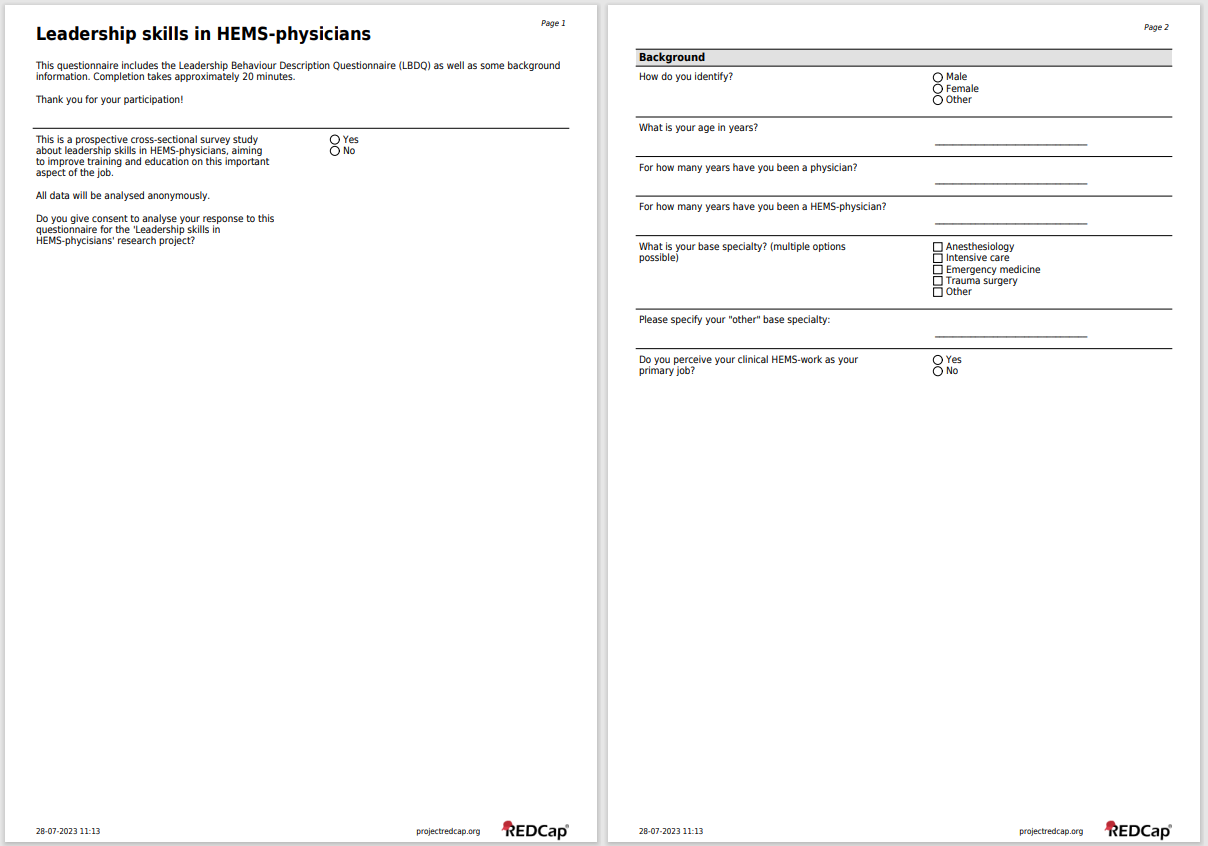


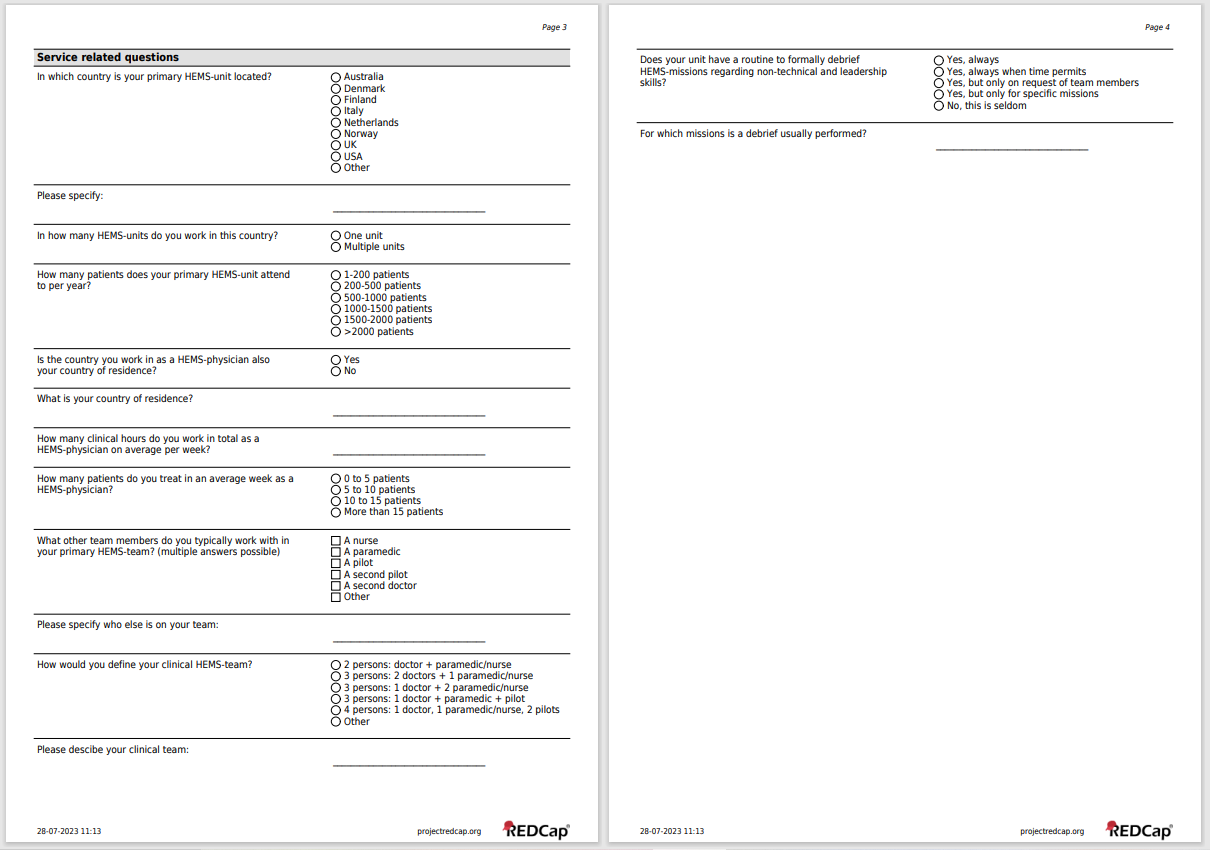


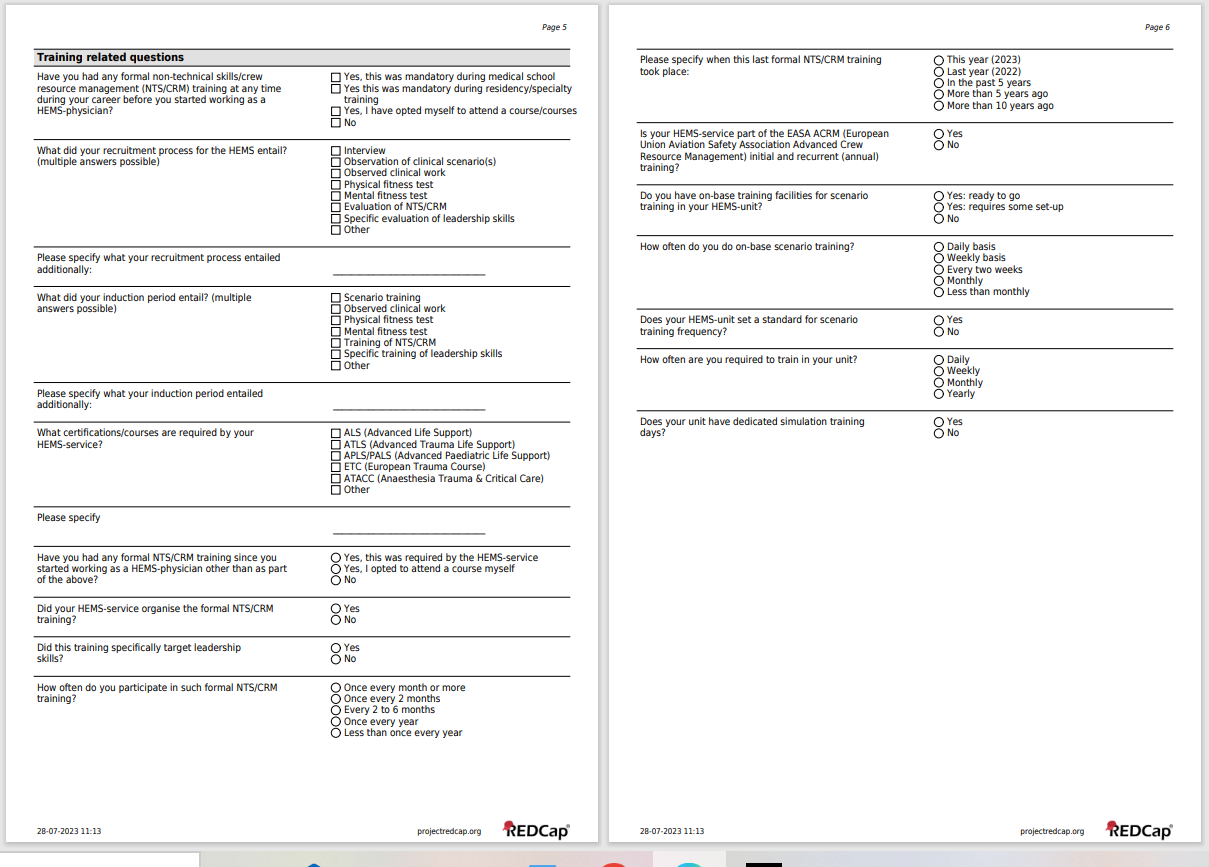


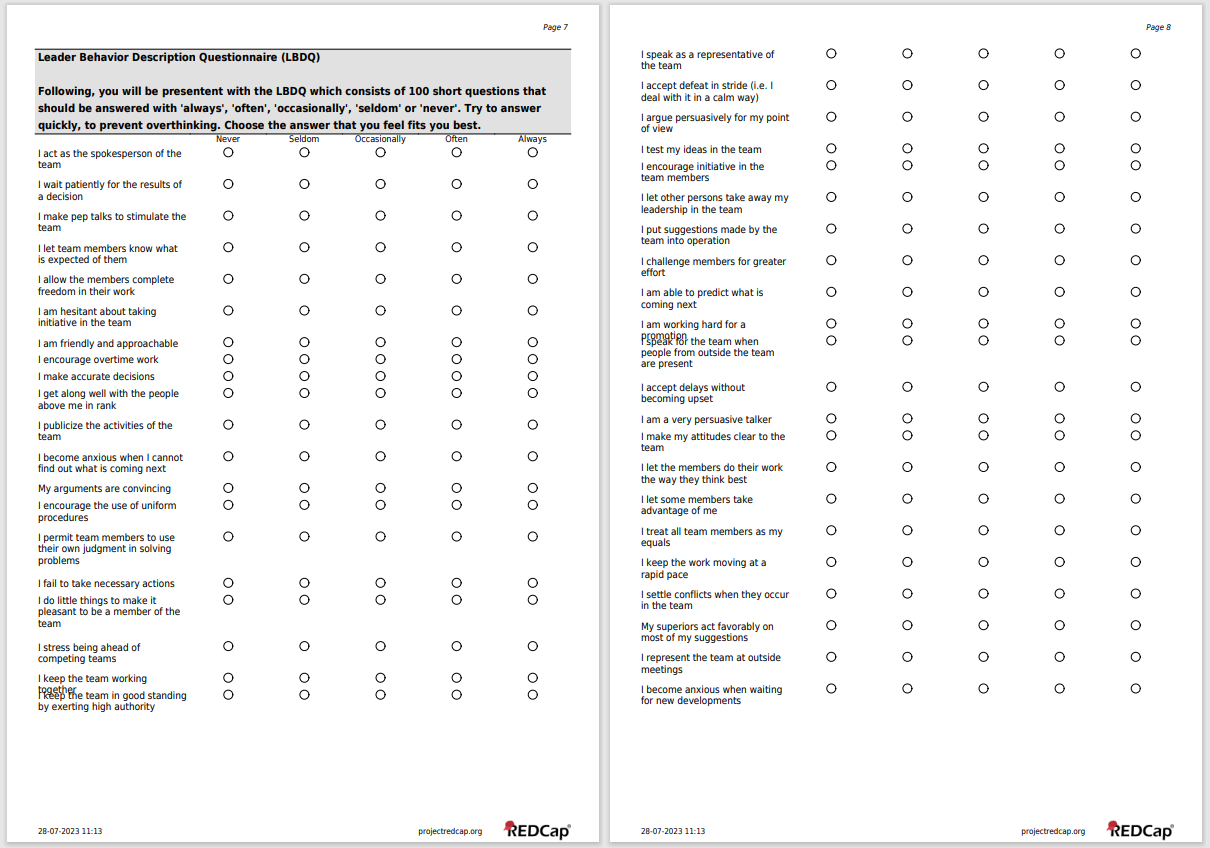


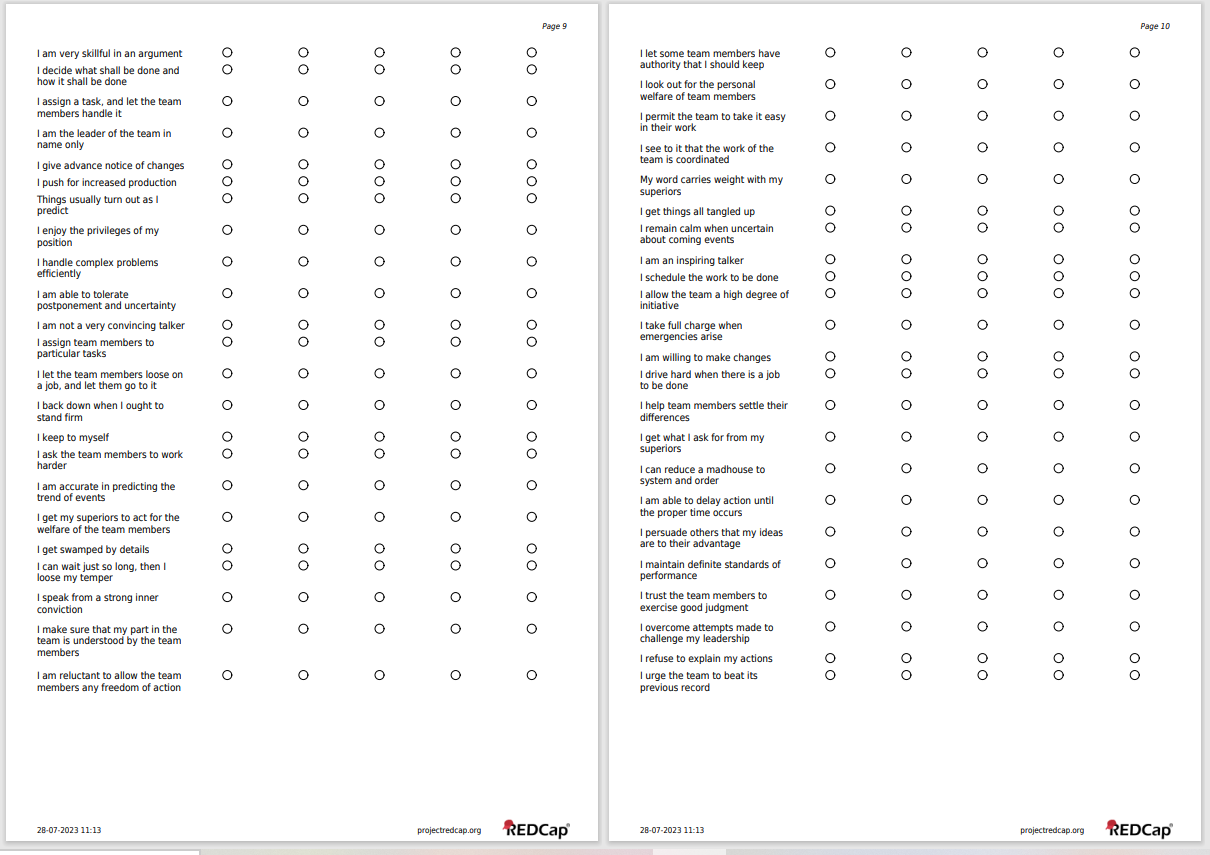


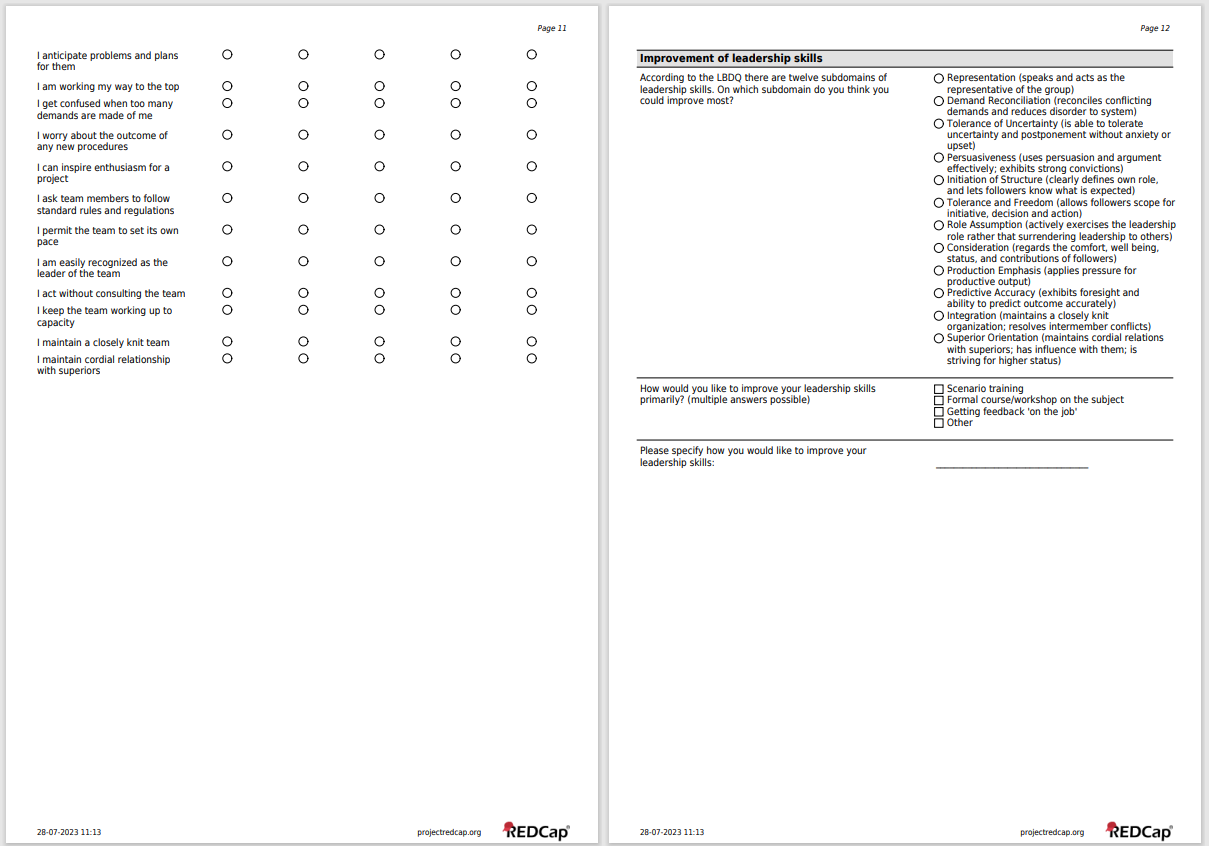

Supplement: Supplementary file 1 — Supplementary Material 1. [file 13049_2024_1221_MOESM1_ESM.docx]

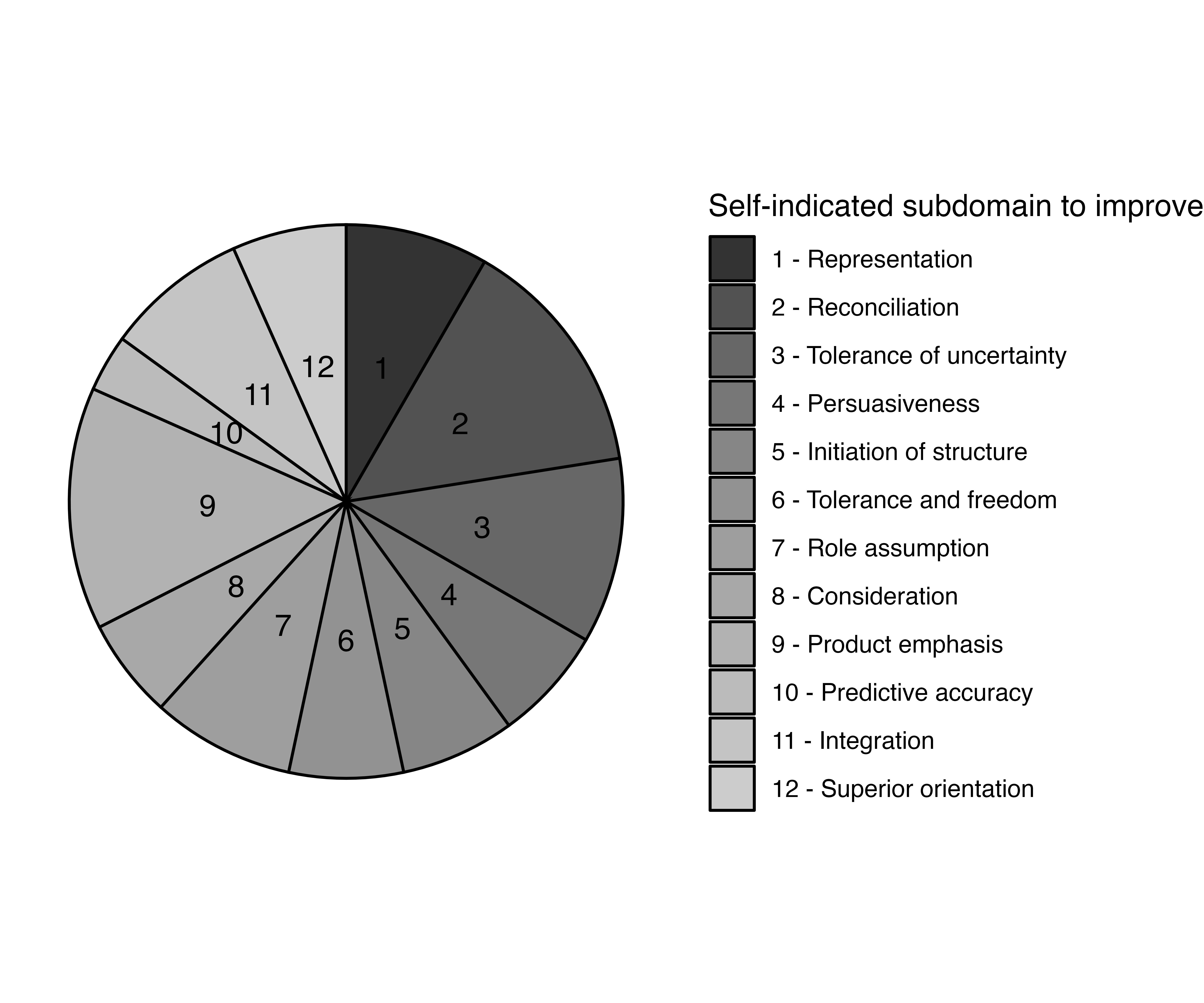

Supplement: Supplementary file 3 — Supplementary Material 3. [file 13049_2024_1221_MOESM3_ESM.jpeg]
